# Optimization of Adhesion in Textile Cord–Rubber Composites: An Experimental and Predictive Modeling Approach

**DOI:** 10.3390/polym17091239

**Published:** 2025-05-01

**Authors:** Merve Pehlivan, Bora Atalik, Sezgin Gokcesular, Sunullah Ozbek, Belma Ozbek

**Affiliations:** 1 Department of Chemical Engineering, Yildiz Technical University, Davutpasa Campus, Istanbul 34220, Turkey; m.pehlivan@brisa.com.tr; 2 Brisa Bridgestone Sabanci Tyre Manufacturing and Trading Inc., Kocaeli 41310, Turkey; b.atalik@brisa.com.tr (B.A.); s.gokcesular@brisa.com.tr (S.G.); 3 Department of Mechanical Engineering, Dogus University, Dudullu Campus, Istanbul 34775, Turkey; sozbek@dogus.edu.tr

**Keywords:** nylon cord, accelerators, textile cord–rubber composites, curing characteristics, adhesion, Box–Behnken, response surface methodology, predictive modeling

## Abstract

The adhesion between rubber compounds and textile cords plays a critical role in determining the overall performance and durability of rubber-based composites, particularly in tire applications. Despite extensive research on adhesion mechanisms, optimizing adhesion through systematic modeling remains challenging due to the complex interactions between rubber formulations, textile treatment, and processing conditions. This study presents an integrated experimental and predictive modeling approach to investigate and optimize the adhesion performance of nylon 6.6 textile cords in rubber compounds. Initially, the effects of different accelerator types—including diphenyl guanidine (DPG), 2,2′-Dithiobis(benzothiazole) (MBTS), N-tert-butyl-2-benzothiazole sulfenamide (TBBS), and N-cyclohexyl-2-benzothiazole sulfenamide (CBS)—on adhesion properties were systematically evaluated. Key parameters such as cure characteristics, Mooney viscosity, and mechanical properties of the rubber compounds were analyzed using a moving die rheometer (MDR), Mooney viscometer, and tensometer. To enhance adhesion performance, a statistical optimization approach based on the Box–Behnken design was employed, focusing on the influence of accelerator, curing agent, and resin contents. The results indicate that an optimized rubber formulation comprising 1.6 phr curing agent, 0.3 phr resin (HMMM), and 0.5 phr accelerator (MBTS) yields the highest adhesion strength. This study provides the first systematic modeling of adhesion between nylon 6.6 textile cords and rubber compounds using response surface methodology (RSM), offering valuable insights into the material design for improved interfacial bonding in tire manufacturing.

## 1. Introduction

Tires are complex, multi-component structures designed to meet specific performance requirements. They consist of various layers, including the inner liner, body ply, bead, sidewall, belt, cap ply, and tread [1]. Among these, the body ply extends from one bead to the other and plays a crucial role in providing structural integrity, maintaining the tire’s shape when inflated, and withstanding mechanical stresses under load and deformation [2]. The body ply is composed of a rubber compound reinforced with either steel or textile cords, which are coated with rubber using a calendering process [3].

Textile cords are widely used in rubber composites to enhance dimensional stability, mechanical strength, and flexibility, allowing the tire to function safely under operational conditions [4,5]. The most commonly used reinforcement materials include nylon, rayon, polyester, and polyester-based textile cords, primarily due to their superior thermal stability during the vulcanization stage of tire manufacturing. The mechanical and adhesion properties of these cords vary depending on their composition and surface treatment [6].

The effectiveness of textile cord reinforcement in tires is directly dependent on adhesion between the rubber matrix and the textile cord [5]. Insufficient adhesion can lead to premature failure due to inadequate load transfer. limiting the structural integrity of the tire [7]. If adhesion is compromised, the textile cord cannot effectively support the tire structure, leading to mechanical failure even under relatively low loads [5].

The primary mechanism responsible for effective adhesion is the formation of covalent bonds and chemical interactions at the rubber–cord interface [8]. However, untreated textile cords typically exhibit poor chemical compatibility with rubber, leading to suboptimal adhesion performance [4]. Several strategies have been developed to enhance adhesion, including: (i) Physical surface treatments: plasma treatment, electron beam irradiation, ultrasonic treatment, and γ-ray radiation; (ii) Chemical modification: ultraviolet (UV)-initiated grafting and chemical grafting to introduce functional groups; (iii) Interfacial enhancement: application of adhesion-promoting dip solutions, such as resorcinol–formaldehyde–latex (RFL), which is widely used in tire manufacturing; and (iv) Rubber formulation adjustments: incorporation of adhesion-promoting resins, fillers, and optimized curing agents [9].

The resorcinol–formaldehyde–latex (RFL) dipping process is a widely used industrial method for improving adhesion between textile cords and rubber compounds [10]. In this process, the resorcinol–formaldehyde (RF) resin in the RFL solution interacts with the textile cord, while the latex component chemically cross-links with rubber during vulcanization [4,5]. This interaction forms a strong interfacial network, enhancing adhesion strength and ensuring flexibility between the rubber matrix and the textile cord [10].

The formulation of the rubber compound significantly influences adhesion properties. Key ingredients affecting adhesion performance include: (i) Rubber type: natural rubber (NR) and styrene–butadiene rubber (SBR) influence mechanical strength and adhesion, (ii) Fillers: carbon black and silica enhance reinforcement but may affect adhesion depending on particle dispersion, (iii) Curing agents: sulfur crosslinking improves durability but must be optimized to prevent over-curing, (iv) Protective additives: antioxidants and antiozonants help maintain adhesion under oxidative and thermal aging conditions, and (v) Resins and accelerators: specific adhesion promoters, such as HexaMethoxyMethylMelamine (HMMM), a methylated melamine–formaldehyde resin, improve bonding by enhancing the crosslinking network at the rubber–cord interface [8,11].

Accelerators play a critical role in vulcanization kinetics, influencing adhesion performance. Different sulfur-based accelerators affect cure rate and crosslink density, affecting adhesion durability and mechanical stability [12].

To investigate and optimize adhesion performance, response surface methodology (RSM) was employed. RSM is a statistical modeling technique used to analyze multi-variable interactions and optimize process parameters [13]. The Box–Behnken design, a widely used RSM technique, was selected for this study due to its efficiency in minimizing the number of experimental runs while maintaining high model accuracy [14,15].

In published literature, there are various studies focused on the understanding of cord–rubber adhesion systems. The fundamental adhesion mechanism of resorcinol–formaldehyde–latex (RFL)-treated cords and rubber was examined in the study conducted by Wennekes et al. [16]. The authors demonstrate the critical interplay between RFL composition, rubber cure chemistry, and processing conditions in achieving robust and durable cord–rubber adhesion. Roshanaei et al. [8] employed a Box–Behnken experimental design to optimize the adhesion between natural rubber/styrene–butadiene rubber (NR/SBR) compounds and polyester cords, systematically investigating the effects of silica, resorcinol, hexamethoxymethyl melamine (HMMM), and CBS/MBTS accelerator content on cord–rubber bonding and mechanical performance. The results indicated that silica prolonged the resorcinol–HMMM reaction to improve adhesion and that the model’s predicted values closely matched the experimental data. Huang et al. [17] developed a novel eco-friendly two-step dipping system for nylon fiber cords by using tea polyphenols and glycerol triglycidyl ether-based coatings, aiming to replace traditional resorcinol–formaldehyde–latex (RFL) adhesives. Piri et al. [18] used a fractional factorial design of experiments (DOE) to optimize the interfacial adhesion between rubber and surface-modified polyester fabric by systematically varying seven process parameters (SBR content, curing temperature, curing time, fabric type, fabric thickness, dip pick-up, and adhesive type), each at two levels. The results showed that curing temperature and fabric surface activation (pre-treatment before dip coating) were the most influential factors governing adhesion. While response surface methodology (RSM) and factorial design have been successfully applied in adhesion studies involving polyester or aramid cords, their application to nylon 6.6 remains notably scarce.

Despite these advancements, cord–rubber adhesion remains a significant challenge, and most existing studies provide only system-specific, empirical insights rather than a general predictive framework. Especially for the adhesion performance of Nylon 6.6–rubber composites, there is a critical research gap in understanding the combined effects of dip formulation, rubber composite formulation, and curing conditions.

The present study aims to bridge this gap by integrating experimental analysis with predictive modeling and statistical optimization to identify key factors and interactions between nylon 6.6 cords and rubber composites. By doing so, it identifies the key parameters and their interactions governing interface bonding and develops a validated predictive model for adhesion performance. Specifically, this study focuses on: (i) investigating the impact of accelerator type on adhesion performance using cord–Rubber adhesion (CRA) tests, (ii) comparing the curing characteristics, viscoelastic properties, and mechanical performance of different rubber formulations using a moving die rheometer (MDR), Mooney viscometer, and tensometer, and (iii) modeling and optimizing the effects of accelerator, resin, and curing agent amounts on adhesion performance using the Box–Behnken statistical design. By analyzing these factors, this research provides new insights into adhesion mechanisms and optimized formulations for textile cord–rubber composites. Moreover, this study represents the first predictive modeling approach for adhesion performance in nylon 6.6–rubber composites using statistical optimization techniques. This approach directly addresses the limitations of prior work and provides a more comprehensive, generalizable basis for improving adhesion in textile cord–rubber composites.

## 2. Materials and Methods

### 2.1. Materials

The styrene–butadiene rubber (SBR) used in this study contains 23.5% bound styrene and exhibits a Mooney viscosity of 52 ML (1 + 4) at 100 °C, contributing to the compound’s processability and mechanical performance. N660-grade carbon black, with a surface area of 35 m^2^/g, serves as a reinforcing filler, enhancing the rubber’s mechanical strength, abrasion resistance, and durability. Stearic acid, functioning as an activator in the vulcanization system, has a melting point of 60 °C and facilitates the dispersion of fillers and curing agents. Phenol–formaldehyde (PF) resins are incorporated as adhesion promoters to improve bonding between the rubber matrix and textile reinforcement. A processing oil with a viscosity of 700 mm^2^/s at 40 °C is utilized to enhance the compound’s plasticity and improve mixing efficiency. Polymerized 2,2,4-trimethyl-1,2-dihydroquinoline (TMQ) serves as an antioxidant, providing protection against oxidative degradation and extending the service life of the rubber compound. 

In this study, four various types of accelerators were investigated to assess their effects on adhesion and curing characteristics: (i) diphenyl guanidine (DPG), (ii) 2,2′-Dithiobis(benzothiazole) (MBTS), (iii) N-tert-butyl-2-benzothiazole sulfenamide (TBBS), and (iv) N-Cyclohexyl-2-benzothiazole sulfenamide (CBS). 

Hexa(methoxymethyl)melamine (HMMM), a methylated melamine–formaldehyde resin, was used as an adhesion promoter, enhancing the interfacial bonding between the rubber matrix and textile cords. 

The curing system included sulfur (with 0.077% heat loss), which facilitates crosslinking reactions, and zinc oxide, which acts as an activator, promoting the formation of a robust cross-linked network. 

The textile reinforcement consists of Nylon 6.6 cords (1400 dtex). These cords were chosen for their high strength, thermal stability, and adhesion compatibility with rubber composites.

### 2.2. Compounding Process

This section presents the rubber compound formulations, incorporating various accelerator types to evaluate their effects on curing characteristics, mechanical properties, and adhesion performance. Each formulation consists of a standard base composition, with variations in the type of accelerator used. The detailed compound recipes are provided in Table 1. The model body ply formulation was derived by integrating insights from two primary sources, Wennekes W. B. [6] and Bridgestone Corporation [19]. The body ply compound was prepared in two sequential mixing stages to ensure uniform dispersion of ingredients and optimal processing characteristics.

#### 2.2.1. Master Batch Stage

In the first mixing stage, the following ingredients were incorporated: natural rubber (NR), styrene–butadiene rubber (SBR), N660 carbon black (CB) as a reinforcing filler, stearic acid as an activator, phenol–formaldehyde (PF) resin as an adhesion promoter, and processing oil to improve mixing efficiency and compound plasticity. The mixing process was carried out in an internal mixer operating at a rotor speed of 80–100 rpm. The mixture was processed until it reached a temperature of 140 °C, at which point it was discharged for cooling and further processing [6,19].

#### 2.2.2. Final Batch Stage

In the second mixing stage, additional components were incorporated into the pre-mixed master batch: polymerized 2,2,4-trimethyl-1,2-dihydroquinoline (TMQ) as an antioxidant, accelerators (DPG, MBTS, TBBS, CBS) to control vulcanization kinetics, hexa(methoxymethyl)melamine (HMMM) resin to enhance adhesion, sulfur as the curing agent and zinc oxide as an activator for the curing system. The final batch was mixed in an internal mixer operating at a rotor speed of 60–70 rpm, and the mixture was discharged once it reached a temperature of 100–110 °C [6,19].

### 2.3. Effect of Accelerator Type on Compound Properties and Adhesion Performance

To evaluate the influence of different accelerator types on the curing characteristics, mechanical properties, and adhesion performance of the rubber compound, four accelerators were studied: diphenyl guanidine (DPG), 2,2′-Dithiobis(benzothiazole) (MBTS), N-tert-butyl-2-benzothiazole sulfenamide (TBBS), and N-Cyclohexyl-2-benzothiazole sulfenamide (CBS). Each accelerator plays a distinct role in the vulcanization process, affecting scorch time, cure rate, crosslink density, and adhesion strength at the rubber–textile cord interface. Table 2 presents the molecular structures of the accelerators used in this study, along with their respective roles in influencing vulcanization kinetics, scorch time, and adhesion performance.

### 2.4. Experimental Design for Adhesion Optimization

To evaluate the effects of accelerator, resin, and curing agent contents on the adhesion performance between rubber composites and textile cords, the Box–Behnken design (BBD) was employed as the experimental design methodology. The Box–Behnken design is a response surface methodology (RSM) approach that efficiently explores the relationships between multiple factors while minimizing the number of experimental runs. This design is characterized by experimental points located at the midpoints of the edges of a multi-dimensional cube, along with central point replicates, ensuring robust statistical modeling. In this study, a three-factor, three-level Box–Behnken design was applied, where the independent variables were accelerator amount (MBTS) (x_1_), resin amount (HMMM) (x_2_) and curing agent amount (sulfur) (x_3_). The experimental conditions were optimized to maximize cord–rubber adhesion strength, as shown in Table 3.

The experimental data obtained from the designed experiments were analyzed using the response surface regression method, employing a second-order polynomial equation to model the relationship between the independent variables and the adhesion response. The general form of the response surface model is expressed as follows:

(1) $$y=f(x_{1}.\;x_{2}.x_{3}.\;\ldots \ldots \;x_{k})$$

where *y* represents the system’s response (cord–rubber adhesion strength), and *x_i_* denotes the independent variables (factors) influencing the response.

To optimize the response variable *y*, a second-order polynomial model was employed in response surface methodology (RSM), given by:

(2) $$y=\beta_{0}+\sum_{i=1}^{k}\beta_{i}x_{i}+\sum_{i=1}^{k}\beta_{ii}x_{i}^{2}+\sum_{i=1}^{k-1}\sum_{J=2}^{k}\beta_{ij}x_{i}x_{j}+\varepsilon$$

where: *x_i_* and *x_j_* are the coded independent variables, *β*_0_ is the intercept term, *β_i_* represents the linear coefficients, *β_ii_* denotes the quadratic coefficients, *β_ij_* represents the interaction coefficients, and *ε* is the random error term [20].

Statistical analysis and regression modeling were conducted using MINITAB 19 software. Based on the experimental design, 15 formulations were evaluated.

### 2.5. Characterization of Rubber Compounds

#### 2.5.1. Rheological Properties

The rheological properties of the rubber compounds were evaluated using a moving die rheometer (MDR 2000, Alpha Technologies, Akron, OH, USA) in accordance with ASTM D5289. The rheometer operated under isothermal test conditions at a temperature range of 25–200 °C and a frequency of 1.667 Hz, maintaining constant strain and frequency. MDR was performed in triplicate to ensure reproducibility. The average values and standard deviations are reported.

The following key parameters were obtained from the rheometer curves: minimum torque (ML), maximum torque (MH), cure extent (MH − ML), scorch time (t_S2_) and optimum cure time (t_90_).

#### 2.5.2. Viscoelastic Properties

The Mooney viscosity and Mooney scorch characteristics were assessed using a Mooney MV 2000E viscometer (Monsanto, Cambridge, UK) in accordance with ASTM D1646. The tests were conducted at an operating temperature range of 25–200 °C and a rotor speed of 0.1–20 rpm to evaluate the viscosity, scorch time, and processability of the rubber compounds. Measurements were performed at 130 °C following ASTM D1646 specifications. Mooney viscosity was performed in triplicate (n = 3) to ensure reproducibility. The average values and standard deviations are reported.

The following key parameters were obtained from the Mooney viscosity curves: Mooney viscosity (ML(1 + 4)) and scorch time (t_5_).

#### 2.5.3. Mechanical Properties

The mechanical properties of the rubber compounds were evaluated after curing using an LPC029 hot press (Fontijne, Niles, MI, USA) under standardized curing conditions. Tensile properties were determined using a T2000 Tensile Tester (Alpha Technologies, Hudson, OH, USA) at a crosshead speed of 300 mm/min at room temperature (23 ± 2 °C). following ASTM D412.

The following parameters were measured: tensile strength (TS), elongation at break (EB), modulus at 100% strain (M100) and 300% strain (M300), And hardness (Shore A). Hardness measurements were conducted using a Zwick/Roell 5109 hardness tester (Zwick/Roell, Ulm, Germany) in compliance with ASTM D2240 (Shore A) standards. Each test was performed four times and the average values and standard deviations were reported.

#### 2.5.4. Adhesion Strength

The adhesion performance of the rubber compounds was evaluated according to ASTM D4776. In this test, a single textile cord was embedded within the rubber matrix, followed by curing under controlled conditions. Adhesion strength was measured both before and after aging at 100 °C for 24 and 96 h. The tests were performed using a universal testing machine (Instron Model 3345, MA, USA), and the adhesion force was expressed in kg/cord. Each test was repeated six times; the average values and standard deviations were reported.

#### 2.5.5. Spectroscopic Characterization: FT-IR Analysis

The changes in the molecular structure of rubber compounds were also detected via attenuated total reflectance-Fourier transform infrared spectrometry (ATR-FTIR) analysis using a Perkin Elmer Spectrum (PerkinElmer Inc., Waltham, MA, USA). Two-model spectrophotometer were between 600 and 4000 cm^−1^.

#### 2.5.6. Morphological Characterization: SEM/EDS Analysis

Surface morphology between textile cords and rubber compounds were investigated by using a scanning electron microscope (SEM. JEOL JSM 6060, JOEL Ltd., Tokyo, Japan). The analysis was performed at an accelerating voltage of 2 kV. Energy-dispersive X-ray spectroscopy (EDS. IXRF, IXRF System Inc., Austin, TX, USA) was additionally utilized to confirm elemental distributions with 10 kV accelerating voltage.

## 3. Results and Discussion

### 3.1. Effects of Accelerator Types

#### 3.1.1. Curing Characteristics of Rubber Compounds

**Figure 1:**
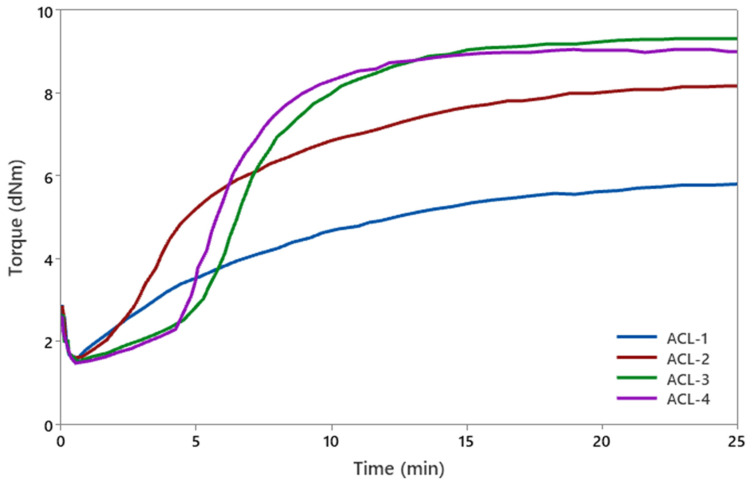
MDR curves of rubber compounds.

The curing characteristics of rubber compounds were evaluated using a moving die rheometer (MDR) at 160 °C for 24 min. In Figure 1, the moving die rheometer (MDR) curves illustrate the curing characteristics of the rubber compounds, including minimum torque (ML), maximum torque (MH), cure extent (MH − ML), scorch time (t_S2_), and optimum cure time (t_90_). Table 4 presents the rheological properties obtained from MDR tests, highlighting the differences in curing behavior based on accelerator type.

The difference between the maximum and minimum torque (MH − ML), referred to as the cure extent, is directly proportional to the crosslink density of the rubber compound. Among the tested formulations, TBBS- and CBS-accelerated rubber compounds (ACL-3 and ACL-4, respectively) exhibited the highest MH − ML values, indicating a greater degree of crosslinking. In contrast, MBTS-based compounds (ACL-2) demonstrated moderate MH − ML values, consistent with previous findings [21]. Scorch time (t_S2_) reflects the processing safety window before the onset of significant crosslinking. Delayed-action accelerators such as TBBS and CBS provided longer scorch times, making them well suited for thick-section products where extended processing time is required. Conversely, accelerators like MBTS resulted in shorter scorch times, favoring fast-curing applications due to their rapid initiation of cross-linking [22]. DPG is used as a secondary accelerator generally and is known as a low scorch time property [23]. However, in this study, DPG exhibits a comparable scorch time due to its use as a primary accelerator with low content [24].

The optimum cure time (t_90_) represents the time required to achieve 90% of the maximum crosslink density. Compounds containing MBTS, TBBS, and CBS as primary accelerators (ACL-2, ACL-3, and ACL-4, respectively) exhibited moderate t_90_ values, which are desirable for achieving efficient crosslinking while minimizing the risk of over-curing [25]. Additionally, the presence of DPG as a primary accelerator with low content resulted in as a higher t_90_ value compared to others [26].

#### 3.1.2. Mooney Viscosity Characteristics of Rubber Compounds

**Figure 2:**
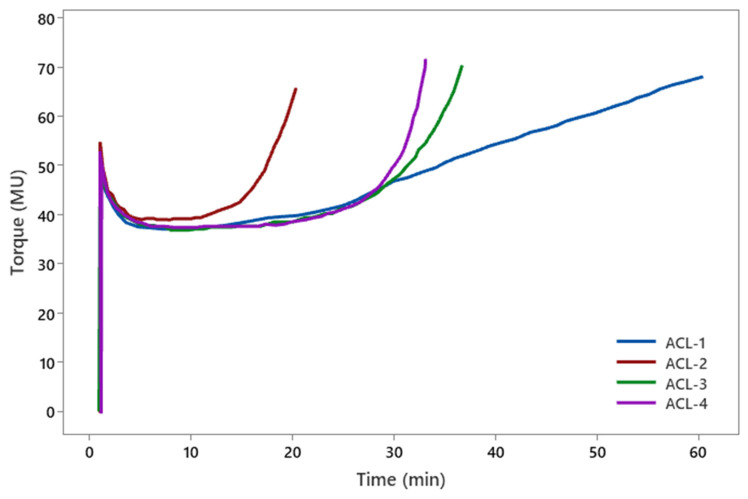
Mooney viscosity curves of rubber compounds.

The Mooney viscosity values of the rubber compounds were measured under specified conditions of time and temperature. Mooney viscosity curves presented in Figure 2 demonstrate the viscoelastic behavior of the rubber compounds, indicating processing characteristics such as flow resistance and scorch time. Table 5 summarizes Mooney viscosity values, scorch times, and flow properties, which are critical for processability and mixing performance.

The Mooney viscosity, denoted as ML(1 + 4), represents the viscosity measured after 4 min of testing following a 1-min preheating period. The values are expressed in Mooney Units (MU), where 1 MU is equivalent to 0.083 N·m.

Scorch time (t_5_) was defined as the times corresponding to viscosity increases of 5 MU. The ML(1 + 4) values for the rubber compounds ranged from 37 to 40 MU. Higher Mooney viscosity values indicate reduced processability due to increased resistance to flow. In this study, the ML(1 + 4) values were found to be relatively consistent across all rubber compounds, suggesting similar processability characteristics.

The scorch time (t_5_) was observed to range between 15 and 26 min, indicating the available processing window before the onset of significant curing. Scorch time is a critical parameter for ensuring safe and controlled processing. Among the tested compounds, ACL-2 exhibited the shortest scorch time, approximately 15 min. Short scorch times can lead to premature curing, posing risks during processing, while excessively long scorch times may prolong production cycles, reducing manufacturing efficiency [27].

#### 3.1.3. Mechanical Properties of Rubber Compounds

The mechanical performance of the cured rubber compounds, including tensile strength (TS), elongation at break (EB), modulus at 100% strain (M100), modulus at 300% strain (M300), and hardness (Shore A), is presented in Table 6. The test specimens were prepared following the ASTM D412 standard.

Tensile strength (TS) represents the maximum stress a rubber compound can withstand under tensile loading before failure, reflecting the material’s overall strength [22]. Elongation at break (EB) measures the extent to which the material can stretch under tensile stress before rupture, indicating its ductility and flexibility [21]. The 100% modulus (M100) denotes the stress required to elongate the material to twice its original length, while the 300% modulus (M300) quantifies the stress needed to stretch the material to three times its original length [21,22].

Following thermal aging, all rubber compounds exhibited increased modulus values, which can be attributed to continued crosslinking during the aging process. The higher M100 and M300 values observed in the ACL-2 compound, which utilizes MBTS as the primary accelerator, indicate a greater crosslink density compared to ACL-1, where DPG is employed as the accelerator [25]. The use of MBTS contributes to enhanced tensile strength by promoting uniform crosslink distribution and mitigating the risk of over-curing (Table 2). Furthermore, MBTS-based compounds exhibit moderate hardness, providing an optimal balance between flexibility and stiffness. This makes them suitable for applications that require both mechanical strength and resilience without compromising elasticity [25].

#### 3.1.4. Cord–Rubber Adhesion Characteristics

**Figure 3:**
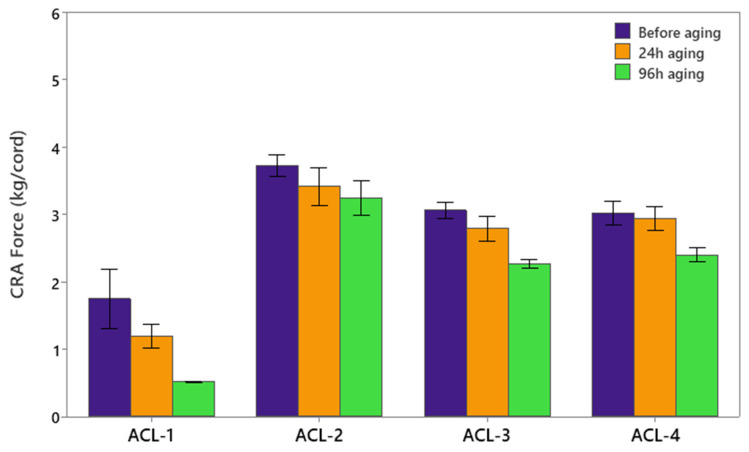
Cord rubber adhesion force (kg/cord) results.

The Cord–rubber adhesion (CRA) test is a standardized method used to evaluate the adhesion strength between reinforcement materials (e.g., textile cords) and rubber compounds [28]. Figure 3 displays the adhesion strength results, showing the effects of different formulations and aging conditions on cord–rubber adhesion performance.

Among the evaluated compounds, ACL-1 exhibited the lowest CRA values, suggesting that DPG, when used as an accelerator, may not promote an optimal crosslink structure required for strong adhesion. In contrast, ACL-3 (containing TBBS as the primary accelerator) and ACL-4 (using CBS) demonstrated intermediate levels of adhesion. The highest adhesion strength was observed in ACL-2, which incorporates MBTS as the accelerator. This superior performance can be attributed to MBTS’s ability to facilitate the formation of a robust crosslink network at the interface between the textile cord and the rubber matrix.

For all rubber compounds, the CRA force decreased after aging for both 24 and 96 h. This reduction in adhesion strength is likely due to thermal and oxidative degradation processes occurring at the cord–rubber interface. Such degradation can compromise the interfacial bonding by reducing the crosslink density within the rubber matrix and at the surface of the textile cord, thereby weakening the overall adhesion performance.

### 3.2. Statistical Analysis of Cord–Rubber Adhesion Performance

All rubber compound formulations, designed based on the principles of design of experiments (DOE), along with their corresponding cord–rubber adhesion (CRA) values, are presented in Table 7. The influence of each formulation parameter on adhesion performance was evaluated using analysis of variance (ANOVA), with the corresponding F-values and *p*-values summarized in Table 8. The effectiveness of each factor was determined by its F-value and *p*-value, where a higher F-value or a lower *p*-value indicates a greater impact on adhesion performance. Based on the ANOVA results, the model shows a statistically significant effect on the CRA response, as indicated by a *p*-value of 0.00 (*p* < 0.05) and an F-value of 23.23. This confirms that the variations in the experimental factors significantly influence the adhesion strength.

Furthermore, the lack-of-fit analysis yielded a non-significant *p*-value (*p* > 0.05), suggesting that there is no substantial discrepancy between the model predictions and the observed data. This indicates that the selected model appropriately fits the experimental results. According to the ANOVA results, the curing agent was identified as the most influential factor affecting the cord–rubber adhesion performance, followed by the accelerator (MBTS) and, subsequently, the resin (HMMM). This ranking highlights the critical roles of the curing system and accelerator type in optimizing interfacial bonding between the textile cord and the rubber matrix.

#### 3.2.1. Main Effect Analysis of Formulation Parameters on Cord-Rubber Adhesion

**Figure 4:**
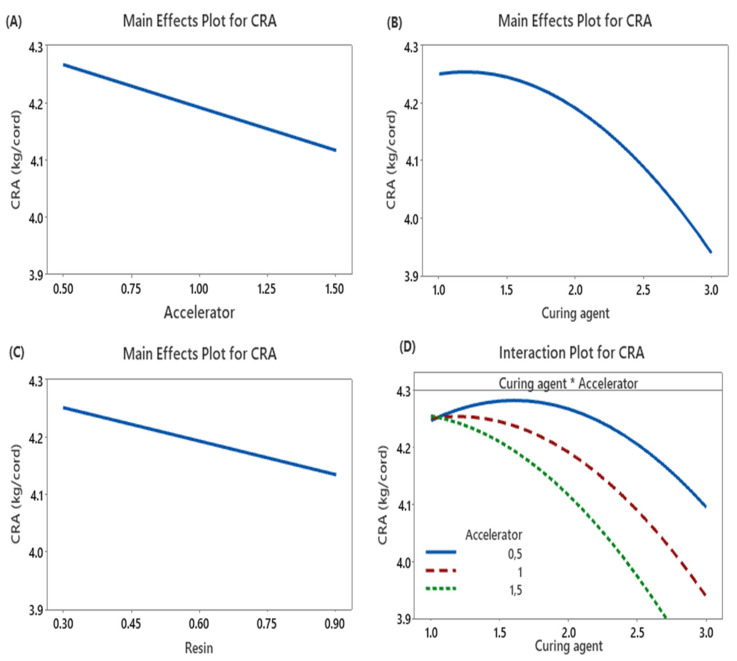
Main effects and interaction effects on adhesion performance (**A**) Accelerator amount, (**B**) Curing agent amount, (**C**) Resin amount, and (**D**) Interaction effects between curing agent and accelerator on adhesion strength.

The main effect plots in Figure 4A–C illustrate the influences of the accelerator, curing agent, and resin content on the adhesion performance between the textile cord and the rubber compound. MBTS, when used as an accelerator, has been reported to provide superior adhesion performance in sulfur-accelerator vulcanization systems [8]. In Figure 4D, the interaction effects between curing agent and accelerator on adhesion strength was represented.

In Figure 4A, increasing the MBTS content leads to a decrease in cord–rubber adhesion. This behavior can be attributed to the disruption of the optimal sulfur-to-accelerator balance within the vulcanization system, which negatively affects crosslink formation at the interface [6]. At lower MBTS concentrations (0.5 phr), the presence of the curing agent (sulfur) positively influences adhesion, likely due to the formation of an effective crosslink network that enhances interfacial bonding (Figure 4D). 

In Figure 4B, the CRA value increases with the curing agent concentration until it reaches a maximum, after which it begins to decline. This behavior can be attributed to an optimal crosslink density at intermediate curing agent levels. At this stage, the formation of sufficient crosslinks enhances interfacial bonding between the rubber matrix and the textile reinforcement. However, at higher MBTS levels, an increase in sulfur content results in reduced adhesion performance (Figure 4D). This decline may be due to over-crosslinking or the formation of less favorable crosslink structures that compromise interfacial strength. 

In Figure 4C, increasing the resin content, specifically hexamethoxymethylmelamine (HMMM), also leads to a reduction in adhesion. This phenomenon can be explained by the excessive loading of HMMM, which disrupts the formation of a stable three-dimensional resin network. Instead, it promotes the formation of linear phenol–formaldehyde resin structures, which are less effective in enhancing adhesion [29].

#### 3.2.2. Two-Dimensional Contour Plot Analysis of Cord-Rubber Adhesion

**Figure 5:**
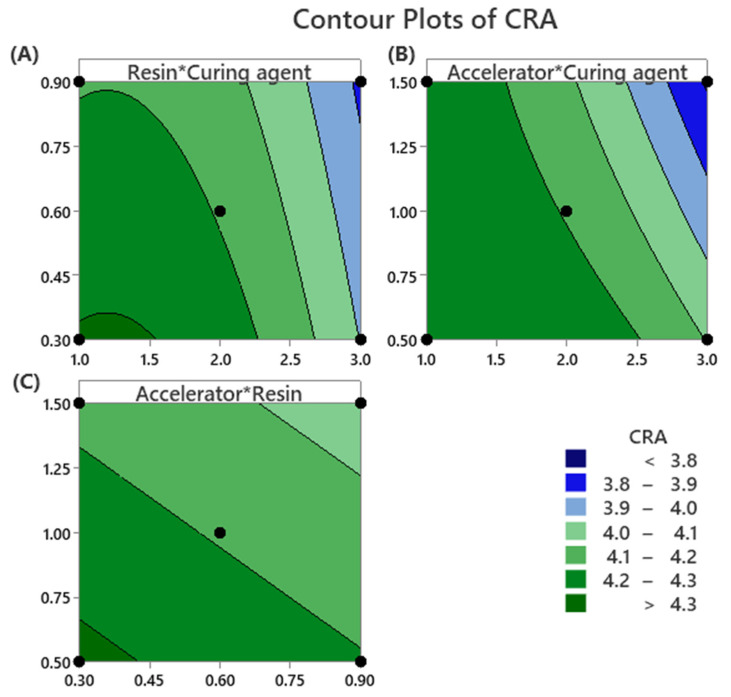
Contour plots for cord–rubber adhesion optimization (**A**) Resin amount and Curing agent amount, (**B**) Accelerator amount and Curing agent amount, and (**C**) Accelerator amount and Resin amount.

The two-dimensional contour plots presented in Figure 5 illustrate the relationship between formulation variables and cord–rubber adhesion (CRA) values, with each contour line representing a constant adhesion response.

In Figure 5A, increasing both the resin (HMMM) and the curing agent (sulfur) concentrations leads to a noticeable decrease in CRA values. This reduction in adhesion performance can be attributed to the potential for over-crosslinking and the formation of rigid, less flexible interfacial networks, which negatively affect bonding strength.

In Figure 5B, the combination of a minimal amount of accelerator (MBTS) and a medium level of curing agent results in the highest CRA values. This suggests that maintaining an optimal balance between the accelerator and curing agent enhances crosslinking efficiency without compromising the flexibility required for strong interfacial adhesion.

In Figure 5C, it is demonstrated that the simultaneous use of minimal amounts of both the accelerator and the resin results in the maximum CRA values under the specified DOE conditions. This observation indicates that lower levels of these components promote favorable interfacial interactions, likely due to reduced interference with the crosslinking network and minimized formation of less effective linear resin structures.

#### 3.2.3. Optimization of Cord-Rubber Adhesion Performance

The cord–rubber adhesion (CRA) values of the rubber compounds were used as the primary criterion for the optimization process. The regression equations derived from the experimental data are presented in Table 9, along with their corresponding R-squared (R^2^) and adjusted R-squared (R^2^(adj)) values. These equations are based on the actual (non-coded) values of the variables. The high R^2^ value of 0.81 indicates that the model explains approximately 81% of the total variation in CRA, which is typically considered indicative of a strong model fit. The adjusted R^2^ value of 0.77 suggests that the model maintains robust explanatory power, even after accounting for the complexity introduced by multiple variables. This demonstrates the model’s reliability in predicting CRA performance under varying formulation conditions. Using the developed statistical models and specified desirability criteria, Minitab 19 software was employed to determine the optimal parameter settings that maximize adhesion performance. In this optimization study, the primary objective was to achieve high adhesion strength between the textile cord and the rubber compound.

**Figure 6:**
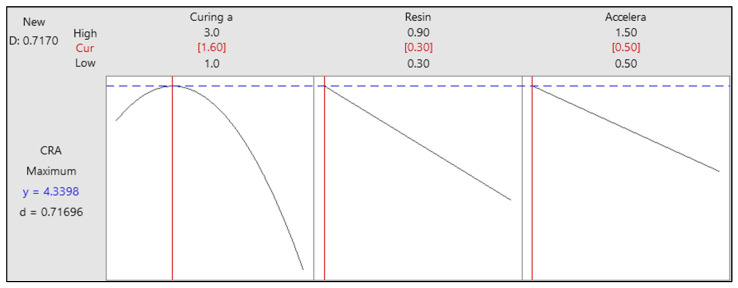
Optimized formulation for maximum cord–rubber adhesion.

Figure 6 presents the optimized values for accelerator, resin, and curing agent amounts, as determined by response surface methodology. According to the optimization results, the optimal rubber compound formulation includes 1.6 phr of the curing agent (sulfur), 0.3 phr of resin (HMMM), and 0.5 phr of accelerator (MBTS). This combination is predicted to yield the maximum CRA value (approximately 4.34 kg/cord) under the defined experimental conditions. The overall desirability score for this optimized formulation is 0.7170, indicating that approximately 72% of the targeted adhesion properties have been successfully achieved through the optimization process. This level of desirability reflects a strong correlation between the optimized formulation and the desired performance outcomes.

### 3.3. Characterization Studies

#### 3.3.1. Spectroscopic Characterization of Rubber Compound

**Figure 7:**
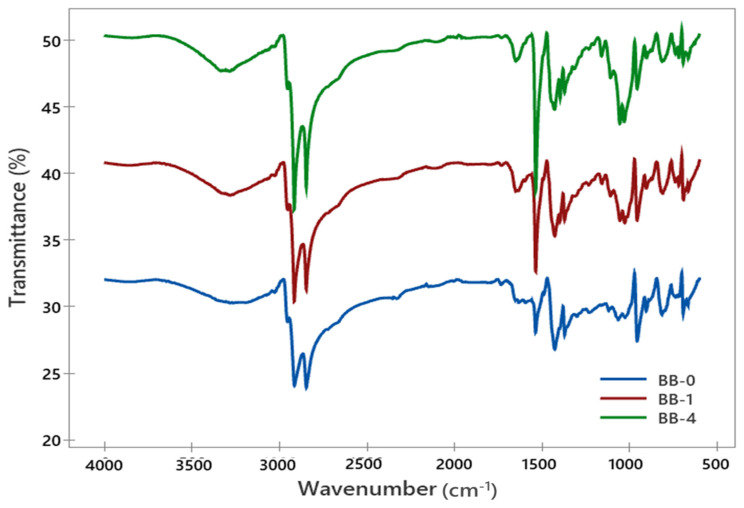
FT-IR spectra of rubber compounds with RSM-predicted optimal formulation (BB-0), maximum adhesion performance (BB-1), minimum adhesion performance (BB-4).

Based on the optimization results obtained via the Box–Behnken design under response surface methodology (RSM), the rubber compound formulations corresponding to the maximum adhesion (BB-1) and minimum adhesion (BB-4) measured by CRA testing, along with the RSM-predicted optimal formulation (BB-0), were selected for FT-IR analysis (Figure 7).

The FT-IR spectra of BB-0, BB-1 and BB-4 are approximately the same, indicating similar functional group compositions. The rubber compounds exhibit two dominant peaks at 3000–2970 cm^−1^ and its corresponding to stretching vibration of −CH (CH_3_ and CH_2_ groups). The peaks at the 1488–1398 cm^−1^ region correspond to chain scission of the −CH group [30]. The 750 and 1000 cm^−1^ peaks relate to C-S and S-O bonds, respectively [31].

#### 3.3.2. Surface Morphology of Rubber Compound

**Figure 8:**
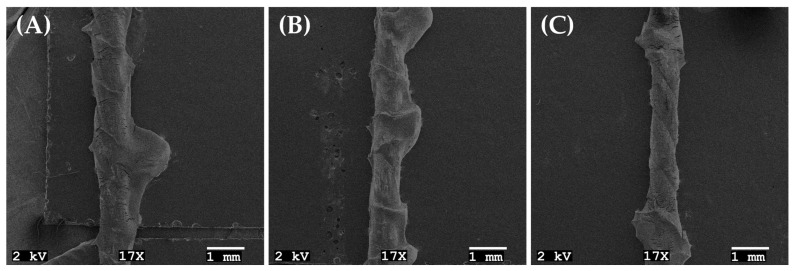
SEM images of surface morphology between textile cords and rubber compounds after CRA testing: (**A**) BB-0, (**B**) BB-1 and (**C**) BB-4.

After CRA testing, the surface morphology between textile cords and rubber compounds was examined by SEM for the formulations exhibiting maximum adhesion (BB-1), minimum adhesion (BB-4) and the RSM-predicted optimal formulation (BB-0) (Figure 8A–C). As shown in Figure 8A,B, BB-0 and BB-1 are completely covered with rubber compounds after CRA testing. The coverage of textile cord with BB-4 compound with the minimum CRA force (3.82 ± 0.15 kg/cord) was weaker than the others (Figure 8C). This phenomenon shows that better coverage of textile cords with rubber compound indicates better CRA force.

**Figure 9:**
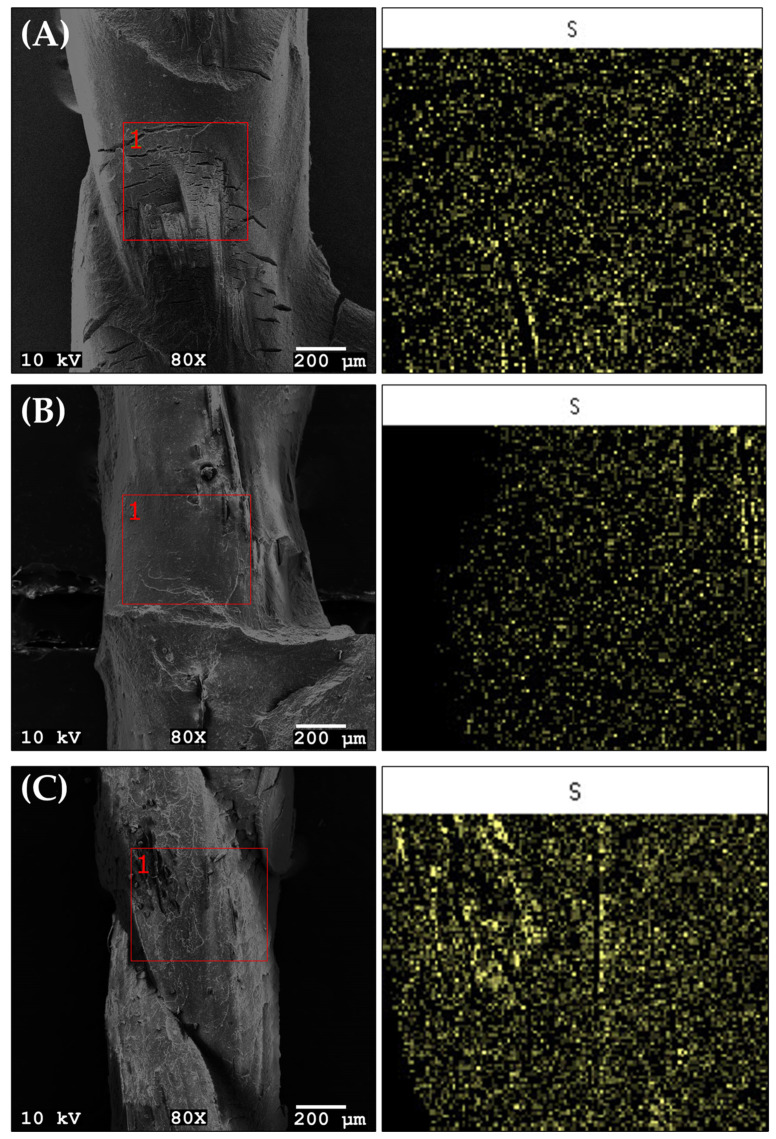
SEM mapping and EDS analysis for sulfur (S) element content between textile cord and rubber compound for: (**A**) BB-0, (**B**) BB-1 and (**C**) BB-4. (The red boxes indicate the selected areas for EDS analysis, and ‘1’ marks the analyzed region.)

Figure 9A–C shows the SEM mapping used to analyze the sulfur (S) element content at the textile cord-rubber compound interface for BB-0, BB-1 and BB-4. The sulfur amounts from textile cord and rubber compound surface after CRA testing were observed by SEM-EDS. The sulfur content at the interface between the textile cord and BB-4 (Figure 9C) is significantly higher compared to the other samples BB-0 and BB-1 (Figure 9A,B). This can be attributed to the increased amount of curing agents in the formulation, which is also consistent with the lower CRA values observed (Figure 9C). The usage of an optimum sulfur amount in rubber compound resulted in strong adhesion.

## 4. Conclusions

In the present study, the adhesion performance between nylon 6.6 textile cords and rubber compounds was systematically examined and optimized using an integrated approach combining experimental analysis and predictive modeling. The key findings of the study are summarized as follows:

- The evaluation of different accelerators identified MBTS as the most effective in enhancing cord–rubber adhesion.
- The effects of varying the amounts of accelerator (MBTS), curing agent (sulfur), and resin (HMMM) on cord–rubber adhesion were examined using the Box–Behnken design method. a robust statistical approach for process optimization.
- Based on the optimization results obtained through the Box–Behnken design, the rubber compound formulation predicted to achieve maximum adhesion performance consists of 1.6 phr of curing agent (sulfur), 0.3 phr of resin (HMMM), and 0.5 phr of accelerator (MBTS).
- The predictive model estimated a maximum adhesion strength of 4.34 kg/cord for the optimized formulation.

Finally, this study represents the first successful predictive modeling of adhesion performance between Nylon 6.6 textile cords and rubber compounds, specifically formulated with the optimized recipe, providing valuable insights into the material design for enhanced interfacial bonding in tire manufacturing.

Future research can expand this model by incorporating additional variables in the rubber formulation that may influence adhesion performance. Specifically, parameters related to resorcinol–formaldehyde–latex (RFL) systems can be integrated to develop a more comprehensive model, potentially improving predictive accuracy for various cord–rubber adhesion applications. Moreover, the effects of surface modification techniques such as plasma treatment, nanocoatings, and functionalized polymer grafting on adhesion performance can be investigated. These approaches could enhance interfacial bonding and durability. Furthermore, the development of a digital twin framework could facilitate real-time simulation and optimization of adhesion behavior, enabling data-driven material design and process control in rubber-based composites.

## Figures and Tables

**Table 1:** Model formulation of rubber compounds with various accelerator types.

Mixing Stage	Component	Content (Phr)			
		ACL-1	ACL-2	ACL-3	ACL-4
Master batch	Natural rubber (NR)	65			
	Styrene–butadiene rubber (SBR)	35			
	Carbon black (CB)	60			
	Stearic acid	1			
	Phenol–formaldehyde resin	1			
	Oil	10			
Final batch	Antioxidant (TMQ)	0.5			
	Accelerator	DPG 1	MBTS 1	TBBS 1	CBS 1
	Resin (HMMM)	0.3			
	Curing agent (Sulfur)	1			
	Zinc oxide	3			

**Table 2:** Chemical structures and functional characteristics of accelerators.

Accelerator Types	Chemical Structures	Functional Characteristics
DPG (Diphenyl guanidine)	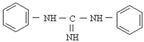	Scorch Slow cure rate
MBTS (2-2′-Dithiobis(benzothiazole))	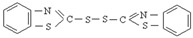	Less scorch Fast cure rate
TBBS (N-tert-butyl-2-benzothiazole sulfenamide)	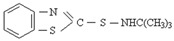	Delayed action Fast cure rate
CBS (N-Cyclohexyl-2-benzothiazole sulfenamide)	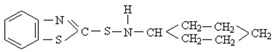	Delayed action Fast cure rate

**Table 3:** Experimental design parameters.

Parameters	Low (−1)	Center (0)	High (+1)
$x_{1}$: Accelerator amount (phr)	0.5	1.0	1.5
$x_{2}$: Resin amount (phr)	0.3	0.6	0.9
$x_{3}$: Curing agent amount (phr)	1.0	2.0	3.0

**Table 4:** Curing characteristics of rubber compounds.

Materials	ML (dNm)	MH (dNm)	MH-ML (dNm)	t_S2_ (min)	t_90_ (min)
ACL-1	1.65 ± 0.04	5.82 ± 0.09	4.17 ± 0.05	5.41 ± 0.13	16.44 ± 0.17
ACL-2	1.69 ± 0.02	8.23 ± 0.06	6.54 ± 0.05	3.26 ± 0.01	14.38 ± 0.13
ACL-3	1.70 ± 0.07	10.26 ± 0.07	8.56 ± 0.06	5.51 ± 0.02	11.84 ± 0.09
ACL-4	1.66 ± 0.02	9.81 ± 0.06	8.15 ± 0.06	4.86 ± 0.01	9.93 ± 0.10

**Table 5:** Mooney viscosity results of rubber compounds.

Materials	ML(1 + 4) (MU)	t_5_ (min)
ACL-1	37.67 ± 0.58	23.62 ± 0.52
ACL-2	39.87 ± 0.06	15.12 ± 0.10
ACL-3	38.63 ± 0.12	25.70 ± 0.32
ACL-4	38.73 ± 0.23	26.12 ± 0.14

**Table 6:** Mechanical properties of rubber compounds.

Materials	Tensile Strength (MPa)	Elongation at Break (%)	100% Modulus (MPa)	300% Modulus (MPa)	Hardness (Shore A)
ACL-1	118.92 ± 4.65	658.17 ± 16.67	8.83 ± 0.17	37.92 ± 0.90	42.00 ± 0.00
ACL-2	176.58 ± 5.05	656.58 ± 17.12	13.58 ± 0.48	64.75 ± 1.01	49.33 ± 0.47
ACL-3	195.33 ± 5.95	609.42 ± 13.16	16.50 ± 0.46	79.42 ± 1.07	51.33 ± 0.47
ACL-4	190.75 ± 6.10	613.00 ± 16.86	15.58 ± 0.48	75.42 ± 1.33	51.67 ± 0.47

**Table 7:** Cord–rubber adhesion results of rubber compounds.

	Content (phr)			Adhesion (kg/cord) (Average)
Run	Curing Agent	Resin	Accelerator	
BB-1	1	0.3	1.0	4.39 ± 0.18
BB-2	3	0.3	1.0	4.14 ± 0.03
BB-3	1	0.9	1.0	4.19 ± 0.20
BB-4	3	0.9	1.0	3.82 ± 0.15
BB-5	1	0.6	0.5	4.05 ± 0.10
BB-6	3	0.6	0.5	4.20 ± 0.06
BB-7	1	0.6	1.5	4.13 ± 0.07
BB-8	3	0.6	1.5	3.83 ± 0.14
BB-9	2	0.3	0.5	4.30 ± 0.14
BB-10	2	0.9	0.5	4.32 ± 0.17
BB-11	2	0.3	1.5	4.19 ± 0.18
BB-12	2	0.9	1.5	4.09 ± 0.13
BB-13	2	0.6	1.0	4.18 ± 0.07
BB-14	2	0.6	1.0	4.13 ± 0.21
BB-15	2	0.6	1.0	4.19 ± 0.14

**Table 8:** Analysis of variance (ANOVA) Results.

Source	DF	Adj SS	Adj MS	F-Value	*p*-Value
Model	10	2.31908	0.231908	23.23	0.000
Blocks	5	1.0006	0.200119	20.05	0.000
Linear	3	1.14843	0.382811	38.35	0.000
$\mathrm{Curing}\;\mathrm{agent}\;\mathrm{amount}\;{(x}_{3})$	1	0.73934	0.739345	74.07	0.000
$\mathrm{Resin}\;\mathrm{amount}\;{(x}_{2})$	1	0.13277	0.132767	13.30	0.001
$\mathrm{Accelerator}\;\mathrm{amount}\;{(x}_{1})$	1	0.20122	0.201222	20.16	0.000
Square	1	0.15211	0.152105	15.24	0.000
$\mathrm{Curing}\;\mathrm{agent}\;\mathrm{amount}\;*\;\mathrm{Curing}\;\mathrm{agent}\;\mathrm{amount}\;{(x}_{3}*x_{3})$	1	0.15211	0.152105	15.24	0.000
2-Way Interaction	1	0.08413	0.084131	8.43	0.005
$\mathrm{Curing}\;\mathrm{agent}\;\mathrm{amount}\;*\;\mathrm{Accelerator}\;\mathrm{amount}\;{(x}_{3}*x_{1})$	1	0.08413	0.084131	8.43	0.005
Error	56	0.55899	0.009982		
Lack-of-Fit	50	0.50636	0.010127	1.15	0.474
Pure error	6	0.05263	0.008772		
Total	66	2.87807			

**Table 9:** Modeling of cord–rubber adhesion values of rubber compounds.

Regression Equation in Uncoded Units	R^2^	R^2^ (adj)
CRA = 4.066 + 0.389 * Curing agent amount − 0.194 * Resin amount − 0.168 * Accelerator amount − 0.096 * Curing agent amount^2^ − 0.159 * Curing agent amount * Accelerator amount	80.58%	77.11%

## Data Availability

Data are contained within the article.
